# Cognitive Domain Impairment and All-Cause Mortality in Older Patients Undergoing Hemodialysis

**DOI:** 10.3389/fendo.2022.828162

**Published:** 2022-03-28

**Authors:** Yidan Guo, Ru Tian, Pengpeng Ye, Xin Li, Guogang Li, Fangping Lu, Yingchun Ma, Yi Sun, Yuzhu Wang, Yuefei Xiao, Qimeng Zhang, Xuefeng Zhao, Haidan Zhao, Yang Luo

**Affiliations:** ^1^ Division of Nephrology, Beijing Shijitan Hospital, Capital Medical University, Bejing, China; ^2^ Division of Injury Prevention and Mental Health National Center for Chronic and Non-communicable Disease Control and Prevention, Chinese Center for Disease Control and Prevention, Beijing, China; ^3^ Department of Nephrology, Beijing Chaoyang Hospital, Capital Medical University, Beijing, China; ^4^ Department of Nephrology, Beijing Shijingshan Hospital, Beijing, China; ^5^ Department of Nephrology, Beijing Huaxin Hospital, The First Hospital of Tsinghua University, Beijing, China; ^6^ Department of Nephrology, China Rehabilitation Research Center Beijing Boai Hospital, Beijing, China; ^7^ Department of Nephrology, Beijing Fuxing Hospital Beijing, Capital Medical University, Beijing, China; ^8^ Department of Nephrology, Beijing Haidian Hospital, Beijing, China; ^9^ Department of Nephrology, Aerospace Central Hospital, Beijing, China; ^10^ Department of Nephrology, Beijing Zhongguancun Hospital, Beijing, China; ^11^ Department of Nephrology, Nankou Hospital of Beijing Changping District, Beijing, China; ^12^ Department of Nephrology, Peking University Shougang Hospital, Beijing, China

**Keywords:** hemodialysis, cognitive impairment, domain, risk factors, mortality

## Abstract

The highly prevalent cognitive impairment in hemodialysis patients is associated with all-cause mortality; however, the role of different cognitive domain impairments in this association is still not clarified. Our objective was to determine the association between cognitive domain impairment and all-cause mortality in elderly adult patients undergoing hemodialysis. We conducted a prospective cohort study including patients from 11 hemodialysis centers in Beijing. Baseline data were collected, and a series of neuropsychological batteries covering 5 domains of cognitive function were included for the assessment of cognitive function. According to the fifth version of the Diagnostic and Statistical Manual of Mental Disorders criteria (DSM-V), the patients were classified as normal, mild, and major cognitive impairment for global and domain cognitive function, then followed up for 1 year. Kaplan–Meier survival analysis was used to compare the difference in the cumulative survival rate in different cognitive domains. A multivariate Cox proportional hazards regression analysis was used to determine the association between global or domain cognitive impairment and all-cause mortality. A total of 613 patients were enrolled, the mean age was 63.82 ± 7.14 years old, and 42.1% were women. After 49.53 ± 8.42 weeks of follow-up, 69 deaths occurred. Kaplan–Meier plots demonstrated a significant association of cognitive impairment in memory, executive function, attention, and language domains with all-cause death. Multivariate Cox regression analysis showed that mild and major impairment of global cognition (HR = 2.89 (95% CI, 1.01–8.34), *p* = 0.049 and HR = 4.35 (95% CI, 1.55–12.16), *p* = 0.005, respectively), executive cognitive domain (HR = 2.51 (95% CI, 1.20–5.24), *p* = 0.014; HR = 3.91 (95% CI, 1.70–9.03), *p* = 0.001, respectively), and memory cognitive domain (HR = 2.13 (95% CI, 1.07–4.24), *p* = 0.031; HR = 3.67 (95% CI, 1.71–7.92), *p* = 0.001, respectively) were associated with all-cause mortality. Combined impairment of 3, 4, and 5 cognitive domains was associated with all-cause mortality [HR = 5.75 (95% CI, 1.88–17.57), *p* = 0.002; HR = 12.42 (95% CI, 3.69–41.80), *p* < 0.001; HR = 13.48 (95% CI, 3.38–53.73), *p* < 0.001, respectively]. We demonstrate an association between the executive and memory cognitive domain impairment and all-cause mortality in hemodialysis patients. Our data suggest that the impairments in these cognitive domains might help in the early identification of hemodialysis patients at risk of death.

## Introduction

Despite the rapid progress in dialysis therapy, the all-cause mortality of the patient undergoing hemodialysis is still kept at a high level (1, 2). A recently published meta-analysis included 86,915 hemodialysis patients in 23 cohort studies evaluated the associated risk factors of all-cause mortality, the results indicated that senior-age, cardiovascular disease, diabetes, anemia, lower level of serum albumin, increased body mass index, and increased serum ferritin, adiponectin, and C-reactive protein were independent risk factors of all-cause mortality in hemodialysis patients (3). However, even the survival time in hemodialysis patients has been prolonged, some potential risk factors for mortality of these patients are still not fully clarified, and identifying those potential risk factors seems to be a priority step in reducing the all-cause mortality in hemodialysis patients (4, 5).

Cognitive impairment is quite common in hemodialysis patients, the prevalence of different extents of cognitive impairment ranged from 70% to 80% (6, 7). Previous cohort studies have demonstrated that global cognitive impairment was an independent risk factor for all-cause mortality, and the death rate increased by1.5- to 2.5-fold in patients with cognitive impairment compared with that in hemodialysis patients with normal cognitive function (8, 9). As the severity of cognitive impairment in cognitive domains was disproportionate in hemodialysis patients (6, 10), we could deduce that the different cognitive domain impairments might have a different relationship with all-cause mortality. Currently, there is still little evidence about the relationship between different cognitive domain impairment and all-cause mortality in hemodialysis patients.

Therefore, we evaluated global and domain cognitive function using a detailed neurocognitive battery of tests and examined whether there is an association between the different cognitive domain impairment and all-cause mortality in a Chinese prospective cohort of patients undergoing hemodialysis.

## Materials and Methods

### Ethics Declaration and Participants

The protocol of this prospective cohort study was approved by the Institutional Ethical Review Board of Beijing Shijitan Hospital, Capital Medical University (Approval No. SJT2016-18); this approved protocol was also authorized by other joining hospitals as a general ethical document. All participants provided written informed consent by themselves or their legal guardians.

We recruited eligible patients from 11 hemodialysis centers in Beijing from April 2017 to June 2017. The inclusion criteria were as follows: (1) age 50 to 80 years, (2) end-stage kidney disease with maintained hemodialysis treatment for a minimum of 3 months, (3) willing to join the study and provide written informed consent, and (4) the patient’s native language was Chinese. The exclusion criteria were as follows: (1) were unable to complete a 90-min cognitive and physical function battery for reasons such as sensory (e.g., visual and hearing) or motor impairment, (2) experienced disturbance of consciousness or were recently diagnosed with psychosis, and (3) had planned kidney transplantation within 6 months of baseline.

Participants’ sociodemographic information and basic characteristics were obtained from patients’ medical charts at the time of enrollment. Medical history, including the history of CVD (a composite of either coronary artery disease and/or peripheral vascular disease), stroke, diabetes, hypertension, and smoking and medication of angiotensin-converting enzyme inhibitors/angiotensin receptor blocker (ACEI/ARB), aspirin, and insulin, were defined by patient history or documentation in the patient’s medical chart. Predialysis blood tests included measurement of the serum levels of hemoglobin, albumin, calcium, phosphate, intact parathyroid hormone (iPTH), C-reactive protein (CRP), and beta2-microglobulin. The estimated glomerular filtration rate (eGFR) was calculated by using the Chronic Kidney Disease Epidemiology Collaboration (CKD-EPI) creatinine equation with an adjusted coefficient of 1.1 for the Asian population, the single-pool Kt/V was calculated from the pre- and postdialysis serum urea nitrogen levels as we applied in the previous study (6).

### Neuropsychological Assessment

According to the recommendation of the fifth version of the Diagnostic and Statistical Manual of Mental Disorders (DSM-V), we evaluated the cognitive function in five cognitive domains (verbal and memory, complex attention, executive function, language, visual-spatial function), and each domain was assessed using validated neuropsychological tests (11, 12).

Neuropsychological tests in our comprehensive battery were selected under the following criteria: psychometric properties, availability of Chinese test norms, maximizing content validity within each domain, minimizing battery duration, and practicality of administration to the clinical practice (6). It included: (1) attention/processing speed, using Symbol Digit Modalities Test (SDMT), and the Chinese-modified version of the Trail Making Test A (TMT-A) (13, 14); (2) executive function, using the Chinese-modified version of the Trail Making Tests B (TMT-B), and a modified version of the Stroop Color-Word Test (SCWT) (15); (3) verbal memory, using the Chinese version of the Auditory Verbal Learning Test (AVLT) for short- and long-delay free recall and complex figure for visual memory (delayed recall test; Chinese version) (16); (4) language, using the Chinese-modified versions of Boston Naming Test (BNT) and Animal Fluency Test (AFT) (17, 18); and (5) visual-space function, using the Rey–Osterrieth Complex Figure (CFT) (19). Depression was assessed using the Hamilton Depression Scale, with scores ranging from 0 to 63 and a score of 7 or above suggested as the optimal cutoff for suspected depression (20). The functional status was evaluated using Lawton and Brody instrumental activities of daily living scales, including 6 basic items and 8 instrumental items (21).

All of the staff for the neuropsychological assessments was trained and certified by the same neuropsychologist before study commencement; all research staffs and patients were native Chinese speakers. To avoid the influence of hemodynamic changes during the dialysis treatment, a neuropsychological assessment was conducted individually on the day after a dialysis session and required approximately 90 min.

### Cognitive Function Classification Algorithm

We classified subjects as having no, mild, or major cognitive impairment using criteria from DSM-V (12). For each cognitive domain impairment, the score of cognitive tests for that domain was calculated and compared with the published norms for Chinese populations. Specifically, scores less than 1.5 standard deviations (SDs) below the mean of the published population norms on all tests indicated no cognitive impairment; scores of 1.50 to 1.99 SDs belonged to mild cognitive impairment, and scores decline to 2.0 or more SDs of the mean of the published norms on at least one test in one domain indicated major cognitive impairment in that domain. Mild impairment in one or more cognitive domains and the cognitive impairment that does not interfere with the capacity for independence in everyday activities was defined as mild global cognitive impairment. Major impairment in one or more cognitive domains and the cognitive impairment that interfere with independence in everyday activities was defined as major global cognitive impairment.

### Study Outcome

The primary outcome after 1 year of follow-up was all-cause mortality. Survival time was defined as the time elapsed from initial study enrollment until death, kidney transplantation, and the end of the follow-up period (June 30, 2018). We obtained the survival status of the patients through periodic medical chart monitoring, as well as contacting each patient’s dialysis unit.

### Statistical Analyses

Descriptive analysis, including proportions, means, and frequencies, was used to define participant characteristics. Continuous variables were expressed as mean with SD or median with interquartile range (IQR); categorical variables were expressed as a number with a percentage. Differences between the groups of no, mild, and major global cognitive impairment were compared using the Chi-square test for categorical variables and one-way ANOVA or Kruskal–Wallis *H* test, as appropriate, for continuous variables. Survival curves were constructed using Kaplan–Meier estimates with comparisons between curves based on the log-rank *χ* ^2^ statistic. The effect of global cognitive impairment, domain cognitive impairment, and the number of domains impaired on the risk of all-cause mortality was quantified by hazard ratios [HRs; with 95% confidence intervals (CIs)] using univariable and multivariable Cox proportional hazard regression analyses. Variables associated at the *p* ≤ 0.10 level with all-cause mortality in unadjusted analyses and potential clinical risk factors were entered into the multivariate regression models as covariates, including age, sex, history of diabetes, hypertension, stroke, and CHD, medication of ACEI/ARB, aspirin and insulin, as well as dialysis vintage, single-pool Kt/V, the levels of hemoglobin, albumin, calcium, phosphate, iPTH, CRP, beta2-microglobulin, and eGFR. When we calculated the effect of cognitive impairment in any domain and the number of domains impaired on the risk of all-cause mortality, the status of cognitive function in other domains was taken as covariates into the multivariate regression models at the power of 0.85. Statistical significance was set at a value of *p* < 0.05. Analyses were performed with SPSS version 21.0 statistical software (SPSS Inc, Chicago, IL, USA).

## Results

### Basic Characteristics of the Participants

We finally included 613 patients in this cohort study (mean age was 63.82 ± 7.14 years, 42.09% were women, 91.35% were married, and only 5.87% had less than 6 years of education). The median hemodialysis vintage was 57 months, and the average treatment session length was 3.81 ± 0.27 h. The baseline features for all patients are displayed in **Table 1**. Patients with cognitive impairment were more likely to be older, have a lower education level, and have a history of diabetes, hypertension, or stroke compared with those with normal cognitive function. In terms of dialysis treatment, patients with cognitive impairment had longer hemodialysis vintage and lower levels of single-pool Kt/V, which reflect lower adequacy of dialysis. On laboratory testing, patients with cognitive impairment had a higher level of serum iPTH.

**Table 1:** Baseline characteristics of the participants with different global cognitive functions.

Characteristics	Total (*n* = 613)	Global cognitive impairment			*p*
		None (*n* = 117)	Mild (*n* = 228)	Major (*n* = 268)	
Age (years)	63.82 ± 7.14	59.29 ± 7.71	63.82 ± 7.14	65.41 ± 7.62	<0.001
Gender (women)	258 (42.1%)	47 (40.2%)	93 (40.8%)	118 (44.0%)	0.695
Marital status					0.773
Single	53 (8.6%)	10 (8.5%)	22 (9.6%)	21 (7.8%)	
Married	560 (91.4%)	107 (91.5%)	206 (90.4%)	247 (92.2%)	
Education level					0.002
<6 years	36 (5.9%)	6 (5.1%)	11 (4.8%)	19 (7.1%)	
6–12 years	408 (66.5%)	63 (53.8%)	169 (74.1%)	175 (65.2%)	
>12 years	169 (27.6%)	48 (41.0%)	48 (21.1%)	74 (27.7%)	
Smoking history					0.783
Never	343 (56.0%)	66 (56.4%)	121 (53.1%)	156 (58.2%)	
Former	186 (30.3%)	34 (29.1%)	76 (33.3%)	76 (28.4%)	
Current	84 (13.7%)	17 (14.5%)	31 (13.6%)	36 (13.4%)	
Alcohol intake					0.226
Never	352 (57.4%)	66 (56.4%)	123 (53.9%)	163 (60.8%)	
Former	233 (38.0%)	45 (38.5%)	90 (39.5%)	98 (36.6%)	
Current	28 (4.6%)	6 (5.1%)	15 (6.6%)	7 (2.6%)	
Medical history					
Diabetes	231 (37.7%)	33 (28.2%)	84 (36.8%)	114 (42.5%)	0.027
Hypertension	545 (88.9%)	95 (81.2%)	203 (89.1%)	250 (93.3%)	0.004
Stroke	100 (16.3%)	6 (5.1%)	38 (16.7%)	56 (20.9%)	0.001
CHD	193 (31.5%)	36 (30.8%)	68 (29.8%)	89 (33.2%)	0.709
Medication history					
ACEI/ARB	410 (66.9%)	70 (59.8%)	163 (71.5%)	177 (66.0%)	0.086
Aspirin	312 (50.9%)	56 (47.9%)	120 (52.6)	136 (50.7%)	0.702
Insulin	178 (29.0%)	31 (26.5%)	68 (29.8)	79 (29.5%)	0.794
BMI (kg/m^2^)	23.60 ± 4.11	24.21 ± 5.78	23.29 ± 3.53	23.60 ± 3.69	0.171
Dialysis vintage (month)	57.00 (24.00, 101.50)	43.00 (12.00, 87.75)	57.00 (20.75, 108.00)	65.00 (32.00, 103.00)	<0.001
Single-pool Kt/V	1.29 ± 0.18	1.37 ± 0.15	1.30 ± 0.19	1.24 ± 0.16	<0.001
Hb (g/L)	11.11 ± 1.46	11.07 ± 1.53	11.11 ± 1.64	11.13 ± 1.27	0.938
Alb (g/L)	39.93 ± 3.18	40.25 ± 2.55	39.99 ± 3.20	39.75 ± 3.38	0.429
CRP (mg/L)	2.60 (1.19, 7.05)	2.37 (1.21, 6.04)	2.79 (1.19, 6.35)	2.70 (1.15, 7.58)	0.807
Calcium (mmol/L)	2.24 ± 0.25	2.21 ± 0.22	2.28 ± 0.25	2.22 ± 0.25	0.018
Phosphate (mmol/L)	1.72 ± 0.65	1.77 ± 0.68	1.67 ± 0.61	1.73 ± 0.67	0.337
iPTH (pg/ml)	271.17 ± 249.85	224.32 ± 200.92	245.97 ± 199.49	313.06 ± 297.41	0.001
beta2-microglobulin (mg/L)	23.84 ± 4.77	24.12 ± 5.61	23.68 ± 4.75	23.84 ± 4.38	0.726
eGFR (ml/min·1.73 m^2^)	9.86 ± 3.25	9.70 ± 3.43	9.80 ± 3.33	9.99 ± 3.10	0.677
ADL total score	20.38 ± 7.30	15.50 ± 1.31	16.54 ± 2.14	25.78 ± 8.07	<0.001
Basic ADL score	7.53 ± 1.81	6.56 ± 0.55	6.90 ± 1.04	8.48 ± 2.19	<0.001
Instrument ADL score	12.85 ± 5.75	8.95 ± 0.85	9.64 ± 1.61	19.29 ± 6.17	<0.001

Data were presented as mean ± SD or median (interquartile range) for continuous variables and number (%) for categorical variables. Abbreviation: CHD, coronary heart disease; ACEI/ARB, angiotensin-converting enzyme inhibitor/angiotensin receptor blockers; BMI, body mass index; Kt/V, an indicator for evaluating dialysis adequacy; Hb, hemoglobin; ALB, albumin; CRP, C-reactive protein; iPTH, intact parathyroid hormone; eGFR, estimated glomerular filtration rate; ADL, activities of daily living.

### Cognitive Impairment in Global and Each Domain

The proportions of patients with cognitive impairment in global cognition and 5 different domains are presented in **Table 2**. There were 228 (37.19%) patients with mild global cognitive impairment, 268 (43.72%) with major global cognitive impairment, and only 117 (19.09%) with normal cognitive function. As for the five domains of cognitive impairment, the highest proportion of the impaired domain was attention, while the lowest was the domain of language. Patients’ cognitive impairment in memory, executive function, and visual-space domains ranged from 40% to 50% (**Table 2**).

**Table 2:** Different proportions of the cognitive impairment in different cognitive domains.

Domains	Cognitive impairment [*n* (%)]			*p*
	Normal	Mild	Major	
Memory	298 (48.61)	159 (25.94)	156 (25.45)	0.048
Executive function	313 (51.06)	111 (18.11)	189 (30.83)	0.031
Attention	211 (34.42)	191 (31.16)	211 (34.42)	0.785
Language	553 (90.21)	50 (8.16)	10 (1.63)	<0.001
Visuospatial function	353 (57.59)	59 (9.62)	201 (32.79)	<0.001
Global cognition	117 (19.09)	228 (37.19)	268 (43.72)	0.0492

### Associations Between Cognitive Function and All-Cause Mortality

#### Global Cognitive Function

**Figure 1:**
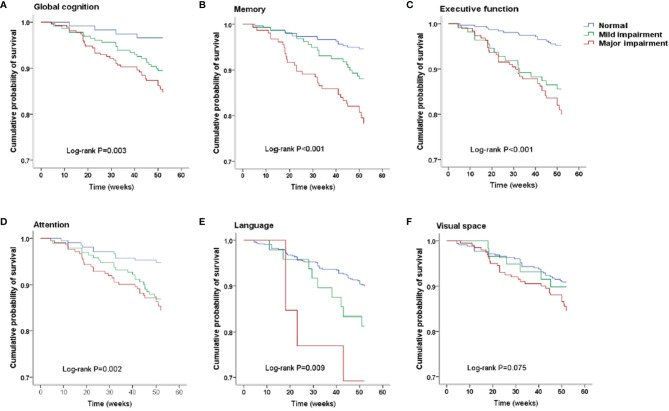
Kaplan–Meier survival curves. **(A)** Survival analysis stratified by global cognition. **(B)** Survival analysis stratified by memory. **(C)** Survival analysis stratified by executive function. **(D)** Survival analysis stratified by attention. **(E)** Survival analysis stratified by language. **(F)** Survival analysis stratified by visual space.

After a median follow-up of 52 weeks, all-cause death had occurred in 69 patients. Specifically, death had occurred in 4 (3.42%) patients in the normal cognition group, in 24 (10.53%) in the mild cognitive impairment group, and 41 (15.30%) in the major group. Kaplan–Meier plots demonstrated an association between cognitive impairment and all-cause death (global log-rank *p* < 0.01; **Figure 1A**). Using Cox proportional hazards models considering the normal cognition group as reference, univariate analysis showed that both mild and major cognitive impairment was associated with a higher hazard for death (HR = 3.19 (95% CI, 1.11–9.18), *p* = 0.032 and HR = 4.75 (95% CI, 1.70–13.25), *p* =0.003, respectively). After multivariable adjustment, the association was reduced somewhat but remained significant [HR = 2.89 (95% CI, 1.01–8.34), *p* = 0.049 and HR = 4.35 (95% CI, 1.55–12.16), *p* = 0.005, respectively].

#### Cognitive Function of Different Cognitive Domains

The number of death for patients with different cognition statuses in 5 domains are displayed in **Table 3**. Patients with cognitive impairment in any domain were more likely to have a higher death rate than those with normal cognition in the same domain. Kaplan–Meier plots demonstrated a significant association between cognitive impairment in memory, executive function, attention and language domain, and all-cause death (global log-rank *p* < 0.01; **Figures 1B–E**) but not in the visual-spatial space domain (global log-rank *p* > 0.05; **Figure 1F**). Univariate Cox regression analysis showed that mild impairment in three of the five domains (memory, executive function, and attention domain) was associated with a higher hazard for mortality, and major impairment in all five domains was associated with a higher hazard for mortality. After multivariable adjustment, the associations of either mild or major impairment with mortality remained significant in memory and executive domains (**Table 3**).

**Table 3:** Risk of death by global and cognitive impairment of 5 domains.

Domains	No. (%) of death			Unadjusted				Multivariate adjusted^b^			
				Mild		Major		Mild		Major	
	Non	Mild	Major	HR (95% CI)^a^	*p*	HR (95% CI)^a^	*p*	HR (95% CI)^a^	*p*	HR (95% CI)^a^	*p*
Memory	16 (2.01)	19 (5.66)	34 (21.79)	2.28 (1.17–4.43)	0.015	4.43 (2.44–8,02)	<0.001	2.13 (1.07–4.24)	0.031	3.67 (1.71–7.92)	0.001
Executive function	15 (4.79)	16 (14.41)	38 (20.11)	3.34 (1.65–6.75)	0.001	4.43 (2.44–8.05)	<0.001	2.51 (1.20–5.24)	0.014	3.91 (1.70–9.03)	0.001
Attention	11 (5.21)	25 (13.09)	33 (15.64)	2.59 (1.28–5.27)	0.008	3.16 (1.60–6.24)	0.001	1.62 (0.77–3.40)	0.207	0.82 (0.34–1.99)	0.667
Language	56 (10.14)	9 (18.75)	4 (30.77)	1.92 (0.95–3.89)	0.068	3.52 (1.28–9.70)	0.015	1.16 (0.55–2.47)	0.694	2.22 (0.74–6.64)	0.156
Visual-space	32 (9.07)	6 (10.17)	31 (15.42)	1.13 (0.47–2.69)	0.790	1.75 (1.07–2.86)	0.027	1.16 (0.46–2.88)	0.757	0.66 (0.33–1.32)	0.238

HR, hazard ratio; CI, confidence interval.

a Hazard ratio from Cox proportional hazard regression analysis; the no-impairment group was used as the reference group.

b Adjusted for age, gender, history of diabetes, hypertension, coronary heart disease and stroke, medication of ACEI/ARB, aspirin and insulin, body mass index, dialysis vintage, Kt/V, hemoglobin, serum levels of albumin, total cholesterol, triglycerides, calcium, phosphate, intact parathyroid hormone, C-reactive protein, beta2-microglobulin, eGFR, and five domain cognitive function.

#### Combined Impairment of Cognitive Domains

**Figure 2:**
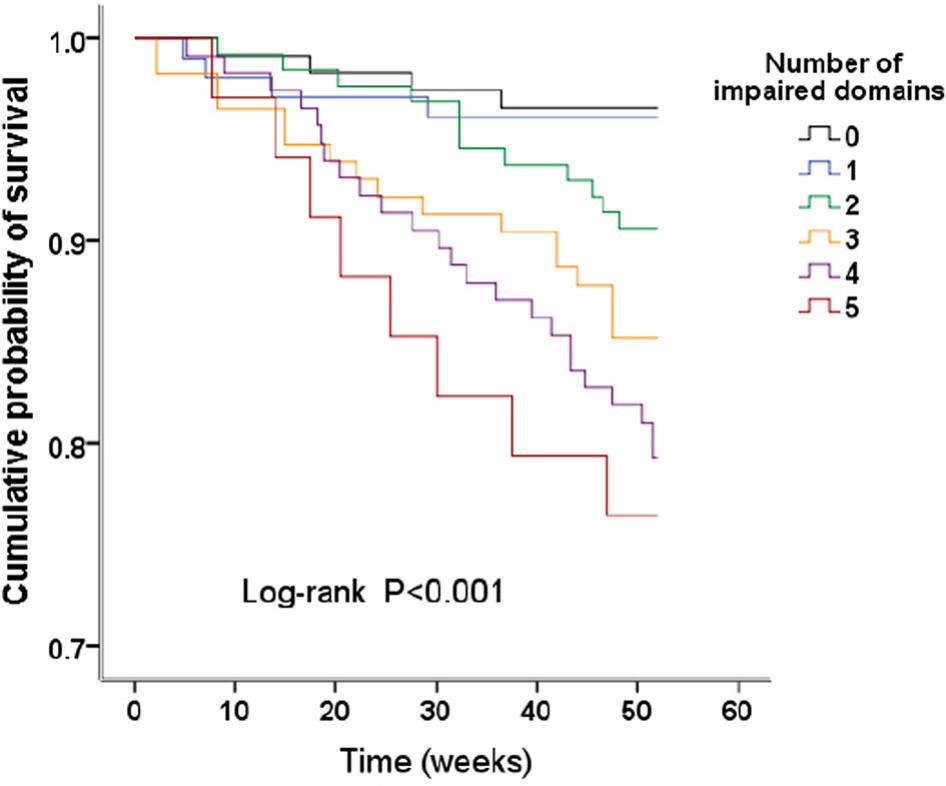
Kaplan–Meier survival curves. Survival analysis stratified by the number of impaired domains.

There were 117 patients with no domains impaired, and 103, 128, 115, 116, and 34 patients with combined injuries in 1 to 5 cognitive domains; the death rates were 4 (3.42%), 4 (3.88%), 12 (9.38%), 17 (14.78%), 24 (20.69%), and 8 (23.53%) for those patients, respectively (**Table 4**). Kaplan–Meier plots demonstrated an association between the number of impaired domains and all-cause death (global log-rank *p* < 0.001; **Figure 2**). Using Cox proportional hazards models considering patients with no domain impaired as a reference, univariate analysis showed that combined impairment of 3, 4, and 5 cognitive domains were associated with a higher hazard for all-cause mortality (HR = 4.59 (95% CI, 1.54–13.69), *p* = 0.006; HR = 6.56 (95% CI, 2.28–18.91), *p* < 0.001; HR = 7.75 (95% CI, 2.34–25.75), *p* = 0.001, respectively). After multivariable adjustment, the association remained significant (**Table 4**).

**Table 4:** The association between the number of impaired cognitive domains and all-cause mortality.

No. of impaired domains	No. of cases (*N*)	No. of death [*n* (%)]	Model 1		Model 2		Model 3	
			HR (95% CI)	*p*	HR (95% CI)	*p*	HR (95% CI)	*p*
0	117	4 (3.42)	1.00 (reference)		1.00 (reference)		1.00 (reference)	
1	103	4 (3.88)	1.15 (0.29–4.59)	0.845	0.95 (0.24–3.80)	0.940	1.07 (0.27–4.28)	0.928
2	128	12 (9.38)	2.80 (0.90–8.68)	0.075	2.56 (0.83–7.95)	0.104	2.97 (0.95–9.26)	0.061
3	115	17 (14.78)	4.59 (1.54–13.63)	0.006	4.04 (1.36–12.02)	0.012	5.75 (1.88–17.57)	0.002
4	116	24 (20.69)	6.56 (2.28–18.91)	<0.001	6.15 (2.13–17.77)	0.001	12.42 (3.69–41.80)	<0.001
5	34	8 (23.53)	7.75 (2.34–25.75)	0.001	6.17 (1.85–20.51)	0.003	13.48 (3.38–53.73)	<0.001

Model 1: unadjusted. Model 2: adjusted for age, gender, history of diabetes, hypertension, coronary heart disease, and stroke, medication of ACEI/ARB, aspirin and insulin, body mass index, dialysis vintage, Kt/V, hemoglobin, serum levels of albumin, total cholesterol, triglycerides, calcium, phosphate, intact parathyroid hormone, C-reactive protein, beta2-microglobulin, and eGFR. Model 3: based on model 2, adjusted for the cognitive function of 5 domains.

## Discussion

In this prospective cohort study, we found that the risk of all-cause mortality increased by 2.5- and 3.9-fold in the patients with mild and major executive domain impairment, and 2.1- and 3.7-fold in those with mild and major memory domain impairment, respectively. Our data also indicated that the patient’s risk of death increased significantly when the number of combined impairment domains was more than 3, which implied that there might be a dose-dependent relationship between combined impairment of cognitive domains and all-cause mortality. As global cognitive impairment has been widely accepted as an independent risk factor of all-cause mortality, the role of cognitive domain impairment in this association is not clear, our data fill this gap and provided evidence of the characteristics of the association between cognitive domain impairment and all-cause mortality among patients undergoing hemodialysis, these results might be a hint in finding the more accurate therapeutic target to reduce the all-cause death in hemodialysis patients.

Cognitive impairment not only decreases the quality of life in hemodialysis patients but also influence the compliance of the patients in respect of diet restriction, fluid control, and medication; these related influence towards the dialysis patients in turn lead the nephrologist to consider the relationship between cognitive impairment and all-cause death. Griva et al. examined this relationship in 145 prevalent hemodialysis and peritoneal dialysis patients; after 7 years of follow-up, they found that cognitive impairment was an independent predictor of all-cause mortality (22). However, some important risk factors of all-cause mortality like serum albumin were not added for the adjustment in the multivariate analysis. Angermann et al. used the Montreal Cognitive Assessment (MoCA) and evaluated the association between global cognitive function and all-cause mortality in 242 maintenance hemodialysis patients. After a median observational interval of 3.54 years, patients with cognitive impairment at baseline of the study had an almost 1.8-fold higher hazard for all-cause mortality after adjustment for commonly known risk factors (23). As the diagnostic criteria of cognitive impairment have been updated in DSM-V and some risk factors of all-cause death have been reported, we evaluated the association of global cognitive impairment and death in a group of Chinese hemodialysis patients. Based on the recommendations in DSM-V and a set of neurocognitive tests with Chinese norms, we found the all-cause death increased to 2.9- and 4.4-fold in patients with mild and major global cognitive impairment, respectively. All these data remind us to pay more attention to inspecting the cognitive function in hemodialysis patients and identifying cognitive impairment among those patients as early as possible.

Global cognitive impairment was made up of the impairment in different domains; however, little information relating to the relationship of different cognitive domain impairment and death was known. A more recent study by Drew et al. found that better executive and memory function were associated with lower mortality in univariate analysis, after adjustment for demographics and dialysis and CV risk factors, the association of executive function and all-cause death remained significant while memory domain impairment became not significantly associated with all-cause death (9). However, this study finished the follow-up in 2013, DSM-V recommendation was not published at that time, several tests were added to the cognitive battery during the study, and this possibly led to less precision within their principal component analysis. Based on the limitation of the previous study, we applied a set of neuropsychological batteries which included the validated neuropsychological tests in five cognitive domains with Chinese norms; after multivariable Cox regression analysis, our results showed that both executive and memory domain impairment was significantly associated with the increased risk of all-cause mortality in hemodialysis patients. Another major result in our study was that combined cognitive impairment was closely related to all-cause mortality; all-cause mortality rates increased from 4.6- to 7.8-fold in the patients with 3 or more cognitive domain impairment. This combined cognitive impairment was also demonstrated by an Italian cohort study; the hazard ratio of all-cause mortality was increased to 2-fold for combined impairment of 3 to 5 domains (24). Our data in cognitive domain impairment, together with previous related studies, indicated that the cognitive domain impairment is disproportionate among individuals undergoing hemodialysis, the risk of all-cause death among those patients was increased when the combined impairment exists in cognitive domains. Depending on the comprehensive neuropsychological battery, our data provided evidence of the association between executive and memory domain impairment and all-cause mortality. We should pay more attention to the function of special impairment domains in the dairy practice.

There are multifactorial aspects about the mechanisms of the associations between cognitive domain impairment and mortality. A newly published meta-analysis including 42 related studies of 3,522 participants showed that the most common impaired cognitive domains of hemodialysis patients were orientation, attention, and executive domains; hemodialysis patients performed better than nondialyzed patients with chronic kidney failure in attention and memory but had poorer memory than the general population (25). Previous studies indicated that the disproportionate cognitive domain impairment might be related to different levels of cerebrovascular disease (26). Our data indicated that the most common impaired domains in this group of dialysis patients were orientation, memory, attention, and executive domains; however, after adjustment for commonly known risk factors, only executive and memory remained significantly associated with all-cause death. The possible explanation of our results is that the executive function is in charge of planning, decision-making, and responding to feedback, patients impaired in this domain can be affected in the adherence to the treatment plans (eg. diet and fluid volume control and medication regimens involved in hemodialysis treatment), which may be particularly associated with adverse outcomes. Similarly, the memory domain is mainly responsible for cued recall and recognition in the process of learning, this is also regarded as a precondition for the executive function and attention (27). We are going to make further exploration about the interrelationship of different domains in the process of cognitive impairment and its influence on the clinical adverse outcomes.

We also have several limitations in this study: first, our study was observational, we cannot adjust all of the residuals and unmeasured confounding. Those potential existing risk factors should be added in the future analysis. Second, some of the hemodialysis patients who were excluded in our study may have more severe cognitive impairment compared with those included participants (e.g., they may have had poorer cognition and poorer health). Hence, we may have underestimated the relationship between some cognitive domain impairment and death. Third, some basic characteristic variables like eGFR and beta2-microglobulin might be influenced by hemodialysis, although those residual kidney functions were regarded to be related to the incidence of cognitive impairments, we did not find them to be associated with mortality in our study, future study should focus on the relationship between cognitive impairments and end-stage renal disease without renal replacement therapy. Lastly, compared with previous studies, we only follow-up the enrolled patients for 1 year, and although we have tried to enlarge our sample size to minimize this limitation, the outcomes may not appear during the follow-up period. Even with those limitations, our study still has some strengths, the evaluation of cognitive function in our study was performed using a comprehensive neuropsychological battery of validated tests with Chinese norms across 5 domains, and the latest criteria of cognitive classification from DSM-V was also applied. We also had a considerable event rate and accounted for currently recognized confounders, including comorbid conditions like cerebrovascular disease and dialysis-related variables.

## Conclusions

We demonstrate an association between the executive and memory cognitive domain impairment and all-cause mortality in a prospective cohort of hemodialysis patients. The tests in our comprehensive neuropsychological battery were chosen according to the recommendation of DSM-V and have all been validated with Chinese norms, and the association between cognitive domain and death was also adjusted with currently recognized confounders, these applied measures ensured the reliability of our data. Our data suggest that the impairments in these cognitive domains might help in the early identification of hemodialysis patients at risk of death. Nevertheless, additional studies are needed to further explore the potential causes of cognitive domain impairment in hemodialysis patients and to try to find a possible interventional target for the prevention of both global and domain cognitive impairment.

## Data Availability Statement

The raw data supporting the conclusions of this article will be made available by the authors, without undue reservation.

## Ethics Statement

The studies involving human participants were reviewed and approved by the Institutional Ethical Review Board of Beijing Shijitan Hospital, Capital Medical University (Approval No. SJT2016-18). The patients/participants provided their written informed consent to participate in this study.

## Author Contributions

YL and YG designed the study. YG, PY, and RT performed the statistical analysis and wrote the manuscript draft. YL, XL, GL, FL, YM, YS, YW, YX, QZ, XZ, HZ, and YL collected data. All authors contributed to data interpretation and editing the final manuscript and approved the final version of the manuscript.

## Funding

This study is supported by a grant from the Beijing Municipal Science & Technology Commission (No. Z161100002616005) and the Capital Health Research and Development of Special (2022-2-2081). The funding sources had no involvement in the study design, data collection, data analysis, the interpretation of data, and writing of the manuscript.

## Conflict of Interest

The authors declare that the research was conducted in the absence of any commercial or financial relationships that could be construed as a potential conflict of interest.

## Publisher’s Note

All claims expressed in this article are solely those of the authors and do not necessarily represent those of their affiliated organizations, or those of the publisher, the editors and the reviewers. Any product that may be evaluated in this article, or claim that may be made by its manufacturer, is not guaranteed or endorsed by the publisher.
